# Ceramide metabolism alterations contribute to Tumor Necrosis Factor-induced melanoma dedifferentiation and predict resistance to immune checkpoint inhibitors in advanced melanoma patients

**DOI:** 10.3389/fimmu.2024.1421432

**Published:** 2024-07-29

**Authors:** Carine Dufau, Matthieu Genais, Elodie Mucher, Benjamin Jung, Virginie Garcia, Anne Montfort, Marie Tosolini, Christopher J. Clarke, Jeffrey A. Medin, Thierry Levade, Jean-Pierre Delord, Nicolas Meyer, Vera Pancaldi, Nathalie Andrieu-Abadie, Bruno Ségui

**Affiliations:** ^1^ Unité Mixte de Recherche Intitut National de la Santé et de la Recherche Médicale (INSERM) 1037, Centre National de la Recherche Scientifique (CNRS) 5071, Université Toulouse III - Paul Sabatier, Centre de Recherches en Cancérologie de Toulouse (CRCT), Toulouse, France; ^2^ Équipe labellisée Fondation Association (ARC), Toulouse, France; ^3^ Stony Brook Cancer Center, and Department of Medicine, Stony Brook University, New York, NY, United States; ^4^ Department of Pediatrics, Medical College of Wisconsin, Milwaukee, WI, United States; ^5^ Department of Biochemistry, Medical College of Wisconsin, Milwaukee, WI, United States; ^6^ Laboratoire de Biochimie, Institut Fédératif de Biologie, Centre Hospitalier Universitaire (CHU) Purpan, Toulouse, France; ^7^ Oncopole Claudius Regaud, Toulouse, France; ^8^ Service d’Oncodermatologie, Institut Universitaire du Cancer (IUCT-O), Centre Hospitalier Universitaire (CHU) de Toulouse, Toulouse, France

**Keywords:** melanoma, TNF, dedifferentiation, sphingolipids, biomarkers, immunotherapy

## Abstract

**Introduction:**

Advanced cutaneous melanoma is a skin cancer characterized by a poor prognosis and high metastatic potential. During metastatic spread, melanoma cells often undergo dedifferentiation toward an invasive phenotype, resulting in reduced expression of microphthalmia-associated transcription factor (MITF)-dependent melanoma antigens and facilitating immune escape. Tumor Necrosis Factor (TNF) is known to be a key factor in melanoma dedifferentiation. Interestingly, accumulating evidence suggests that TNF may play a role in melanoma progression and resistance to immunotherapies. Additionally, TNF has been identified as a potent regulator of sphingolipid metabolism, which could contribute to melanoma aggressiveness and the process of melanoma dedifferentiation.

**Methods:**

We conducted RNA sequencing and mass spectrometry analyses to investigate TNF-induced dedifferentiation in two melanoma cell lines. *In vitro* experiments were performed to manipulate sphingolipid metabolism using genetic or pharmacologic alterations in combination with TNF treatment, aiming to elucidate the potential involvement of this metabolism in TNF-induced dedifferentiation. Lastly, to evaluate the clinical significance of our findings, we performed unsupervised analysis of plasma sphingolipid levels in 48 patients receiving treatment with immune checkpoint inhibitors, either alone or in combination with anti-TNF therapy.

**Results:**

Herein, we demonstrate that TNF-induced melanoma cell dedifferentiation is associated with a global modulation of sphingolipid metabolism. Specifically, TNF decreases the expression and activity of acid ceramidase (AC), encoded by the *ASAH1* gene, while increasing the expression of glucosylceramide synthase (GCS), encoded by the *UGCG* gene. Remarkably, knockdown of AC alone via RNA interference is enough to induce melanoma cell dedifferentiation. Furthermore, treatment with Eliglustat, a GCS inhibitor, inhibits TNF-induced melanoma cell dedifferentiation. Lastly, analysis of plasma samples from patients treated with immune checkpoint inhibitors, with or without anti-TNF therapy, revealed significant predictive sphingolipids. Notably, the top 8 predictive sphingolipids, including glycosphingolipids, were associated with a poor response to immunotherapy.

**Discussion:**

Our study highlights that ceramide metabolism alterations are causally involved in TNF-induced melanoma cell dedifferentiation and suggests that the evolution of specific ceramide metabolites in plasma may be considered as predictive biomarkers of resistance to immunotherapy.

## Introduction

Treatments for patients with metastatic melanoma have recently been revolutionized by the development of immunotherapies targeting immune checkpoints such as CTLA-4 and PD-1 with specific inhibitors (Immune checkpoint inhibitors, ICI). Despite their success, a significant proportion of patients either do not benefit or experience tumor relapse within two years of treatment induction (1). Moreover, most patients develop major immune-related adverse events (irAEs). In patients who are refractory to corticotherapy, some irAEs, such as colitis, can be treated with a bolus of infliximab, an anti-Tumor Necrosis Factor α (TNF) antibody (2). Whether TNF blockade affects the anti-melanoma immune response triggered by immunotherapies in patients remains to be determined.

Our previous work highlighted that TNF impairs anti-melanoma immune responses by inducing cell death of activated CD8+ T lymphocytes in a TNF Receptor 1 (TNFR1)-dependent manner (3). TNF, produced in melanoma tumors following ICI, is involved in the resistance to anti-PD-1 antibody in mice (4). Accordingly, anti-TNF antibody treatment significantly increases the anti-tumor effect triggered by anti-PD-1 antibody (4). Notably, Melero and co-workers showed that anti-TNF compounds not only improve ICI efficacy but also their tolerability in preclinical cancer models, including the B16F10-Ova model (5). Finally, in one of our clinical studies, we evaluated the safety and tolerability of combining an anti-TNF agent, either infliximab or certolizumab, with anti-CTLA-4 (ipilimumab) and anti-PD-1 (nivolumab) antibodies in advanced melanoma patients. The first results indicate that both tritherapies are safe, with promising signs of efficacy, especially in the certolizumab cohort (6).

TNF plays a dual role in oncoimmunology, either acting as an anti-cancer factor, or behaving as an immunosuppressive cytokine (7, 8). Despite TNF having been first identified as a cytotoxic soluble factor, mounting evidence indicates that TNF facilitates the accumulation and/or biological activity of immunosuppressive cells such as regulatory T lymphocytes (Tregs), regulatory B lymphocytes (Bregs) as well as myeloid-derived suppressor cells (MDSC) (7, 8). Furthermore, in a mouse model of adoptive transfer of specific T cells, TNF was found to directly impact tumor cells and lead to the establishment of an epithelial-to-mesenchymal transition (EMT)-like process called melanoma dedifferentiation (9).

Melanoma dedifferentiation is characterized by a decrease in the expression of melanocyte antigens by tumor cells leading to a less efficient anti-tumor immune response. The transcription factor MITF (microphthalmia-associated transcription factor), the key regulator of melanocyte differentiation is central in this process (10). Indeed, decreased expression of MITF and a concomitant increase in stem factors have been associated with resistance to treatment in melanoma (11, 12). Moreover, melanoma dedifferentiation is also involved in the metastatic spread as it confers invasive properties to tumor cells (10). The establishment of this mechanism by TNF could contribute to the aggressiveness of melanoma.

TNF is also a potent modulator of sphingolipid metabolism. Sphingolipids are sphingoid base-containing lipids, which contribute to membrane composition and regulate cell signaling. TNF activates the so-called sphingomyelin-ceramide pathways as a consequence of sphingomyelinase (SMase) activation (13, 14). Ceramide can be further metabolized to sphingosine 1-phosphate (S1P), *via* the sequential activation of ceramidases and sphingosine kinases (SK) (15, 16). sphingosine kinase 1 (SK1) and S1P are key players of TNF signaling in melanoma cells (15). Moreover, high levels of SK1 expression in melanoma cells participate in resistance to ICI (anti-PD-1 or anti-CTLA-4) in preclinical melanoma models. Thus, SK1 inhibition potentiates the regression of melanoma upon ICI therapy in mice (17). Conversely, we showed that neutral SMase 2 is frequently downregulated in human melanoma samples and mouse melanoma cell lines. Re-expressing this enzyme in mouse melanoma cells enhances ceramide production and response to ICI (18). Interestingly, a growing body of evidence indicates that sphingolipids might contribute to melanoma dedifferentiation *via* the regulation of MITF. Indeed, the acid SMase elicits MAPK activation and proteasomal degradation of MITF in melanoma (19). Moreover, acid ceramidase (AC) has been identified as a direct transcriptional target of MITF and a key enzyme in the invasive to proliferative phenotypic switch of melanoma cells (20).

Herein, we evaluate the impact of ceramide metabolism changes triggered by TNF in melanoma cell dedifferentiation. We show that TNF-induced AC inhibition and glycosphingolipid production contributes to accumulation of ceramide metabolites and melanoma cell dedifferentiation. Moreover, we observed an increase in glycosphingolipids in plasma of melanoma patients who did not respond to ICI.

## Materials and methods

### Cell culture

The human primary melanoma WM35 cells were from Dr. M. Herlyn (The Wistar Institute, Philadelphia, PA). The human metastatic 451Lu cells were provided by Dr. S. Tartare-Deckert (Université Nice Sophia-Antipolis, Inserm, Center Méditerranéen de Médecine Moléculaire, Nice, France). The human metastatic A375 cells were from ATCC (LGC). The WM35 cells were cultured in medium containing 80% (v/v) filtered MCDB153 medium (Sigma-Aldrich) complemented with 17.86 mM NaHCO3 and adjusted to pH 7.4, 20% (v/v) Leibovitz’s L-15 medium (Gibco, Thermo Fisher Scientific) supplemented with 1.68 mM CaCl2, 5 μg/mL human insulin solution (Sigma-Aldrich) and 2% Fetal Bovine Serum (FBS). The A375 and 451LU cells were grown in DMEM medium supplemented with 10% FBS. All cell lines were regularly tested for mycoplasma contamination. Melanoma cells were authenticated by STR profiling (Eurofins Genomics).

### Cell treatments

Cells were seeded at Day 0, and then treated at Day 1 with or without 50 ng/mL human recombinant TNF (Peprotech). When indicated, cells were treated at Day 1 with 6 µM of Eliglustat (Sanofi Genzyme), 5 µM of Fumonisin B1 (Sigma-Aldrich), and 10 µM of C2-ceramide (Avanti Polar Lipids 860502P). For transfection experiments with siRNAs, a solution of Opti-MEM containing RNAimax lipofectamine (Invitrogen, dilution 1/100) and 10 nM of siRNA was prepared and left for 20 min at room temperature. The cells were then seeded with this solution. siCTRL (5’GUACCGCACGUCAUUCGUAUCdTdT3’), siASAH1–1 (5’AAUCAACCUAUCCUCCUUCAGdTdT3’) and siASAH1–2 (5’CACGAUUAACUGUGAAAUGUAdTdT3’) were from Eurofins. siASAH1–3 was from ThermoFisher (Catalog number: AM16708 ID:14389).

### RNA-Seq analyses

WM35 were seeded at Day 0 and then treated in reverse kinetics for 6, 24, 48 and 72 hours with 50 ng/mL TNF. Four independent experiments were performed. Parental A375 cells were seeded and then treated with or without 50 ng/mL TNF for 24 hours. Four independent experiments were performed. Alternatively, WM35 were transfected with siCTRL or siASAH1–1 72 hours before RNA extraction. Two independent experiments were performed with siASAH1–1 carried out in duplicate. RNAs were extracted using a Qiagen RNA extraction kit (RNeasy Plus mini kit) and then reverse transcribed using the RT iScript enzyme (Biorad). RNA sequencing was performed by the technology cluster of the Cancer Research Center of Toulouse. FASTQ files were aligned using bowtie2 and Homo sapiens transcriptome reference GRCh38 (v97), the raw count matrix was generated using RSEM (v1.2.25) (21). Differential analysis was performed using the DESeq2 package of R (22). Significantly differentially expressed genes were defined as having logFC<1 or logFC>1 and corrected p value <0.05. Their enrichments of signaling pathways were calculated using AutoCompare ZE (23) with the Gene Ontology C5, KEGG, Reactome and H databases. The Sample Enrichment Score (SES) was calculated using AutoCompare_SES (24).

### Definition of differentiation state signatures from RNA-Seq data

Cell differentiation state gene signatures were recovered from literature (see **Supplementary Materials** and **Methods** for details). For each gene signature, we created a score, computing the geometric mean of the expression of genes. We then computed the Spearman correlation (25) of these scores across 173 human melanoma tumors public RNA-Seq expression profiles (26–28) and TCGA SKCM dataset (471 tumor samples).

We performed hierarchical clustering (complete link of corrplot package) on the (signatures) differentiation scores correlation matrix and observed 3 clearly separated clusters which we named Dedifferentiated, Transitory, and Differentiated. We then calculated the average of differentiation scores in each cluster to obtain 3 values for each one of the 644 expression profiles (471 from TCGA SKCM and 173 from Gide Riaz and Liu melanoma datasets), which we denoted as the Dedifferentiated, Transitory and Differentiated features, respectively. We then calculated Spearman correlations between these features and enrichment scores of each dataset for the Gene Ontology term ‘sphingolipid biological process’(calculated using the GSVA functions from the gsva package (29).

### Transcription factor activities

To estimate transcription factor (TF) activities, we used DoRothEA (30), which is a database of TF-target interactions (regulons). TF activities were estimated using analytic Rank-based Enrichment Analysis (aREA) from the Viper R package (31), as part of the DoRothEA R package. aREA computes a normalized enrichment score for each regulon based on the average ranks of its targets. The DoRothEA database ranks regulons with a confidence level (from A to E). We filtered out only the E level to get more insight from the database, leaving us with 361 regulons. To identify TFs that have significantly differential activities across conditions, we used the msviper function, from the VIPER package, which performs MAster Regulator INference Analysis.

### Data visualization

Several packages were used for data visualization: FactoMineR (32) and factoextra (33) for all PCA plots; Corrplot (34) for the correlation plot of dedifferentiation signatures; ggplot2 (35) for Venn diagrams with ggvenn, and all boxplots; ComplexHeatmap (36) for heatmaps.

### Plasma sphingolipid content in advanced melanoma patients treated with ipilimumab and nivolumab

Ceramide metabolites in the plasma of advanced melanoma patients treated with ipilimumab and nivolumab (bi-therapy) or ipilimumab, nivolumab and anti-TNF (certolizumab or infliximab) enrolled in the clinical trials MELANFα (NCT03348891) and TICIMEL (NCT03293784) were measured by LC/MS. MELANFα is a translational proof-of-concept, open-label, prospective, multicenter trial, aiming to identify the clinical markers and/or biomarkers associated with therapeutic response to immune checkpoints inhibitors, in patients with advanced melanoma. The first results of this study were communicated at the ESMO congress in September 2022 and are submitted for publication. In this clinical trial, between September 2018 and November 2020, 25 patients treated with ipilimumab and nivolumab were included. TICIMEL is a phase 1b clinical trial in advanced melanoma patients the primary objective of which is to evaluate the safety and security of co-administering ipilimumab, nivolumab and anti-TNF (certolizumab or infliximab). In this clinical trial, between January 2018 and January 2022, 33 patients were enrolled. The first results of TICIMEL were published (6) and updated results were communicated at the ESMO congress in September 2022. In both MELANFα and TICIMEL, clinical responses were evaluated using RECIST V1.1 criteria at week 12 post-treatment induction.

### Machine learning model predicting therapy response based on sphingolipid score

Based on the plasma concentration of 78 distinct ceramide metabolites, we created a sphingolipid score from the log2 fold changes of these sphingolipid concentrations under treatment based on the predictive power evaluated by the Boruta algorithm. The top 8 sphingolipids were used to calculate this score. All patients gave their informed consent to be enrolled in this trial and have their blood samples analyzed for research purposes.

### Statistical analysis

All statistical analyses were performed using GraphPad Prism 9 software. Results are expressed as mean ± SEM, and group comparisons were performed with a paired T Test for comparison of 2 groups, or a One-way ANOVA test for comparison of experiments that consisted of ≥ 3 groups. A p value lower than 0.05 was considered statistically significant (*, P < 0.05; **, P < 0.01; ***, P < 0.001; ****, P < 0.0001).

## Results

### TNF-induced melanoma cell dedifferentiation is associated with changes in sphingolipid metabolism

**Figure 1:**
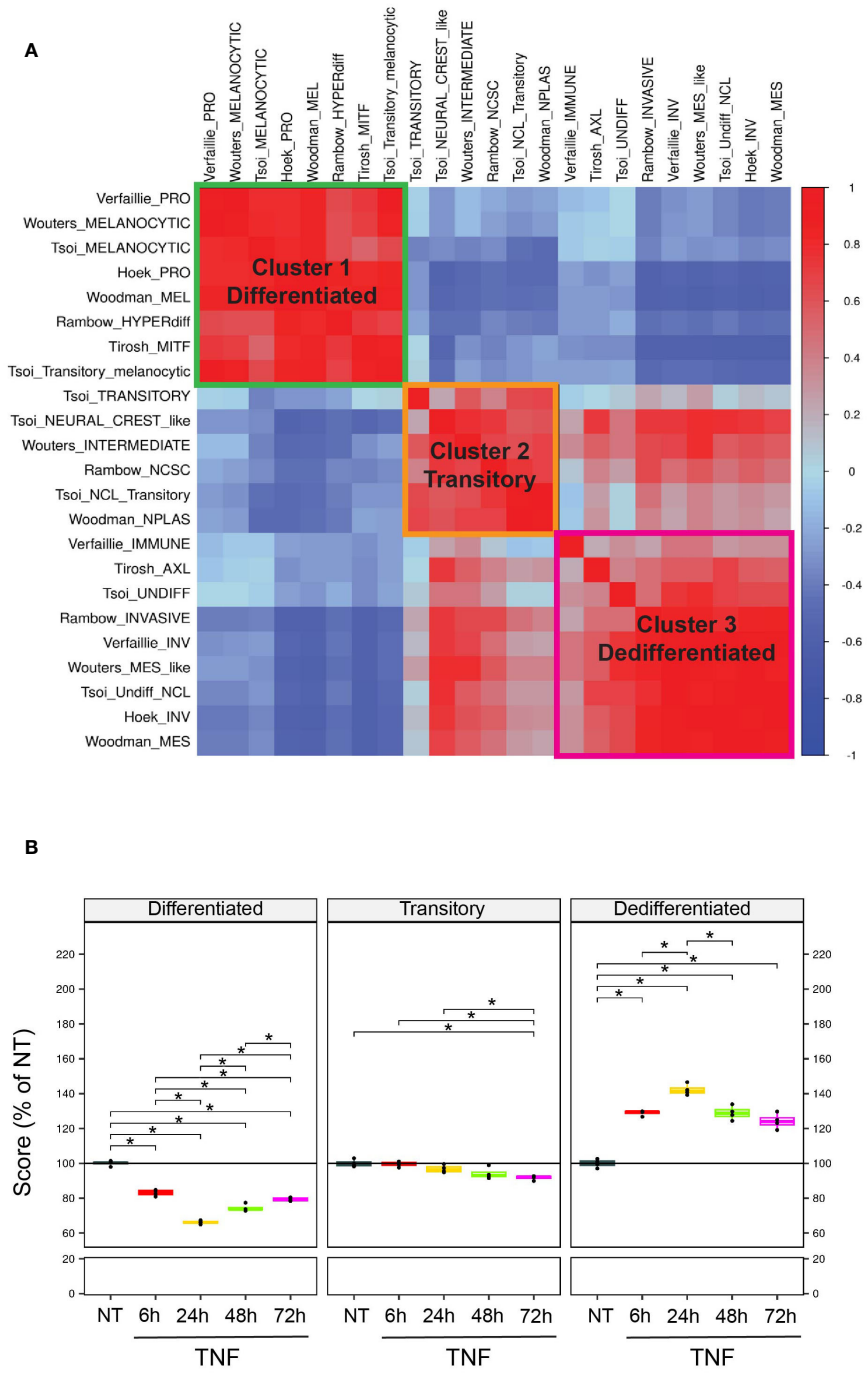
Scoring method to monitor differentiation states in melanoma from RNA sequencing data. **(A)** Correlation plot (Spearman) across various dedifferentiation signatures (see methods) on public melanoma patient cohorts. Signatures were clustered using unsupervised hierarchical clustering. Blue=negative correlations; Red=positive correlations. **(B)** WM35 melanoma cells were untreated (NT) or treated with 50 ng/mL TNF for 6, 24, 48 or 72 hours, and then analyzed by RNA-Seq (n=4). Plots represent the evolution of the scores upon TNF treatment compared to control. *p<0.05.

To characterize the impact of TNF on the dedifferentiation process, we leveraged different gene signatures from the literature associated with dedifferentiation (see method). Correlation analyses on 4 publicly available datasets of tumor biopsies from melanoma patients, including the TCGA melanoma dataset, revealed 3 main clusters of highly positively correlated gene signatures (**Figure 1A**). Thus, three main states of melanoma differentiation were considered: differentiated, transitory and dedifferentiated. Importantly, those signatures exhibited a minimal gene overlap (**Supplementary Figure 1**), indicating that each signature captured a specific aspect of the melanoma differentiation states. To better describe TNF-induced dedifferentiation, we merged the different gene signatures that were positively correlated, based on hierarchical clustering, and generated three features to describe the differentiated, transitory and dedifferentiated melanoma states. Then, to evaluate the impact of ceramide metabolism changes in TNF-induced melanoma cell dedifferentiation, we incubated the WM35 human primary melanoma cell line with TNF for 6, 24, 48 and 72 hours. Global transcriptomic analysis by RNA sequencing (RNA-Seq) demonstrated that TNF increased the expression of genes belonging to “Hallmark TNF signaling via NFKB” in TNF-treated WM35 melanoma cells (**Supplementary Figure 2**). In addition, TNF triggered WM35 cell dedifferentiation, as shown by a shift in the value of our differentiation feature scores from differentiated to dedifferentiated (**Figure 1B**). TNF treatment led to an early increase in the dedifferentiation score at 6 hours post-treatment, reaching its maximum effect at 24 hours, followed by a progressive decrease at 48 and 72 hours (**Figure 1B**). Accordingly, the differentiation score decreased significantly with a minimum value at 24 hours. TNF-induced dedifferentiation was confirmed at the protein level by flow cytometry analyses. Indeed, TNF promoted the upregulation of the stemness factor NGFR at the cell surface of WM35, while concurrently decreasing the expression of the melanocytic marker Melan-a (**Supplementary Figure 3A**). The impact of TNF on melanoma dedifferentiation was not restricted to the primary melanoma cell line WM35. Indeed, the metastatic melanoma cell line 451Lu, when treated with TNF, also underwent a dedifferentiation process, as evidenced by RT-qPCR on a set of 5 transcripts representative of the differentiation status (**Supplementary Figure 3B**). This dedifferentiation phenomenon was further confirmed at the protein level by flow cytometry, revealing a significant decrease of Melan-a expression at 48 and 72 hours, accompanied by a trend showing NGFR upregulation at all times (**Supplementary Figure 3C**).

**Figure 2:**
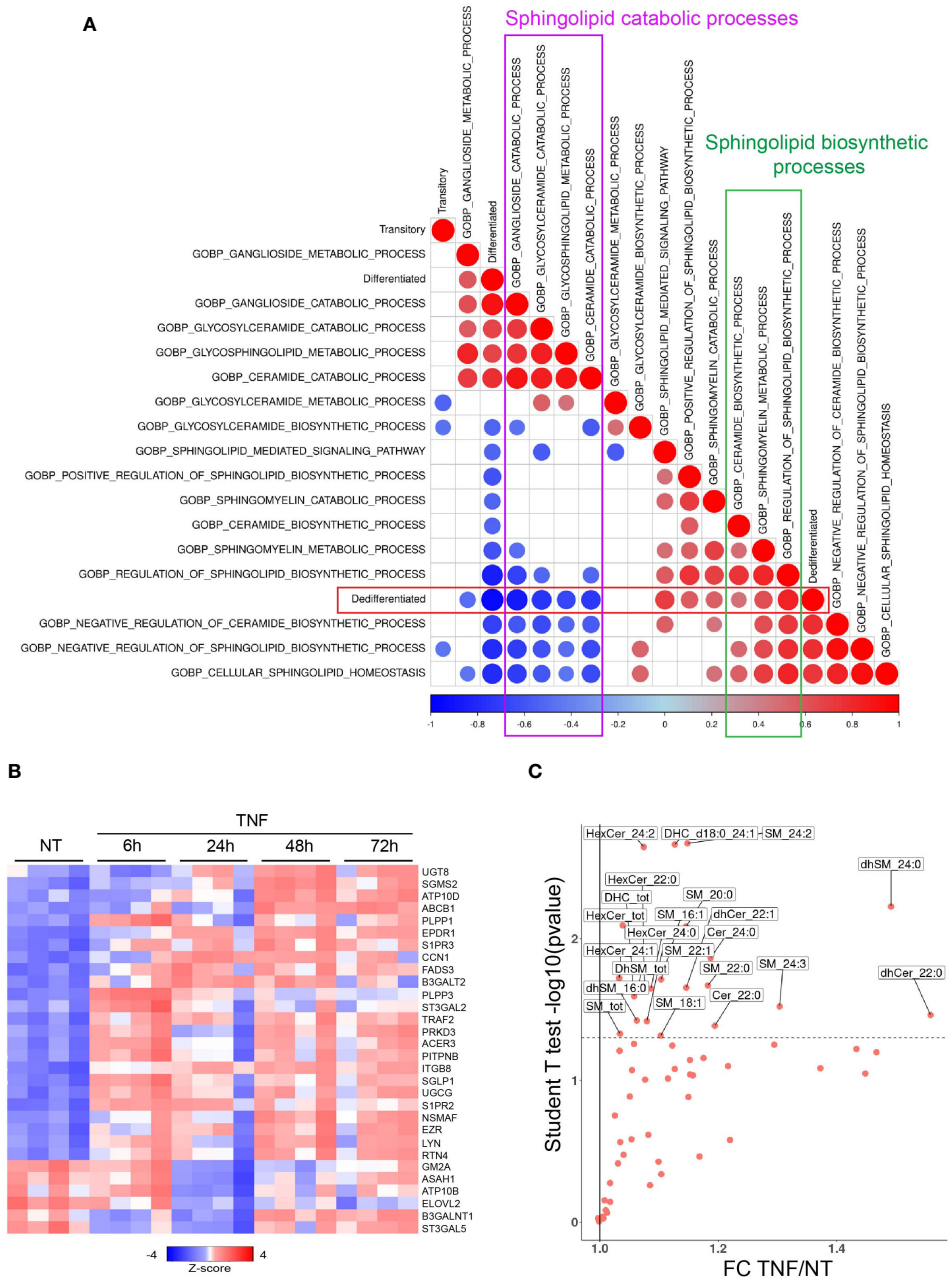
TNF-induced melanoma dedifferentiation is associated with a global accumulation of sphingolipids. **(A)** Spearman correlations between the melanoma differentiation scores and sphingolipid-related biological processes (GO; gsva activities) from WM35 melanoma cells treated or not with TNF as described in the legend to **Figure 1B**. Color represent the correlation value and size the pvalue (only pval<0.05 represented). **(B)** Heatmap showing the expression of ceramide metabolism genes significantly modulated by TNF in WM35 melanoma cells (RNAseq, n=4). **(C)** Sphingolipid content as analyzed by Mass Spectrometry of WM35 untreated (NT) or treated with 50 ng/mL TNF for 48 hours.

Among the pathways regulated by TNF signaling, sphingolipid-related biological pathways, as defined by Gene Ontology (GO), were correlated with melanoma dedifferentiation. In fact, the dedifferentiation state was negatively correlated with the catabolic processes for gangliosides (r=-0.86, p=10^-6^), glycosylceramide (r=-0.75, p=10^-4^) and ceramide (r=-0.73, p=10^-4^), while showing a positive correlation with sphingolipid biosynthesis (r=0.84; p=10^-6^) (**Figure 2A**). With a similar kinetic of the effect of TNF on the differentiation state of melanoma cells, extended analysis of the GO pathways indicated an increased activation of the ceramide and glycosylceramide biosynthetic process, along with a decreased activation of sphingolipid catabolic pathways upon TNF treatment, with the maximum effect observed at 24 hours and then a progressive decline at 48 and 72 hours (**Supplementary Figure 4A**). To explore this further, we examined the expression of genes related to ceramide metabolism upon TNF treatment. These genes exhibited the most differential expression at 24 hours post-TNF treatment when compared to the control group (**Figure 2B**). Among the 24 genes with increased expression across timepoints, we identified *UGCG* and *SGMS2*, encoding glucosylceramide synthase and the sphingomyelin synthase 2, respectively. Conversely, we observed decreased expression of 6 genes including *ASAH1*, encoding the acid ceramidase (AC) (**Figure 2B**). Similar with observations in WM35 cells, TNF triggered the decrease of *ASAH1* expression in 451Lu cells, concomitant with an increase in *UGCG* expression (**Supplementary Figure 4B**).

To further evaluate the impact of TNF signaling on ceramide metabolism, we performed mass spectrometry analyses of lipids extracted from WM35 cells treated with TNF for 24, 48 and 72 hours. The most significant changes were observed at 48 hours post-TNF treatment, manifesting as an increase of various ceramide metabolites, including some (dihydro)sphingomyelins, (dihydro)ceramides, monohexosylceramides and dihexosylceramides (**Figure 2C**). To investigate the role of ceramide metabolism in melanoma cell dedifferentiation induced by TNF treatment, we incubated the WM35 melanoma cell line with exogenous ceramides (**Supplementary Figure 4C**) or with an inhibitor of ceramide synthesis (**Supplementary Figure 4D**). Under our experimental conditions, exogenous ceramides significantly reduced Melan-a expression (**Supplementary Figure 4C**), suggesting that ceramide, or its metabolites, might be involved in melanoma cell dedifferentiation. Conversely, incubating melanoma cell lines in the presence of Fumonisin B1 (FB1), which inhibits *de novo* sphingolipid synthesis, partially prevented the loss of Melan-a expression upon TNF treatment (**Supplementary Figure 4D**).

Collectively, these data indicate that TNF-induced melanoma cell dedifferentiation is associated with the accumulation of ceramide metabolites, which likely contribute to melanoma cell dedifferentiation.

### TNF-induced acid ceramidase inhibition participates in melanoma cell dedifferentiation

**Figure 3:**
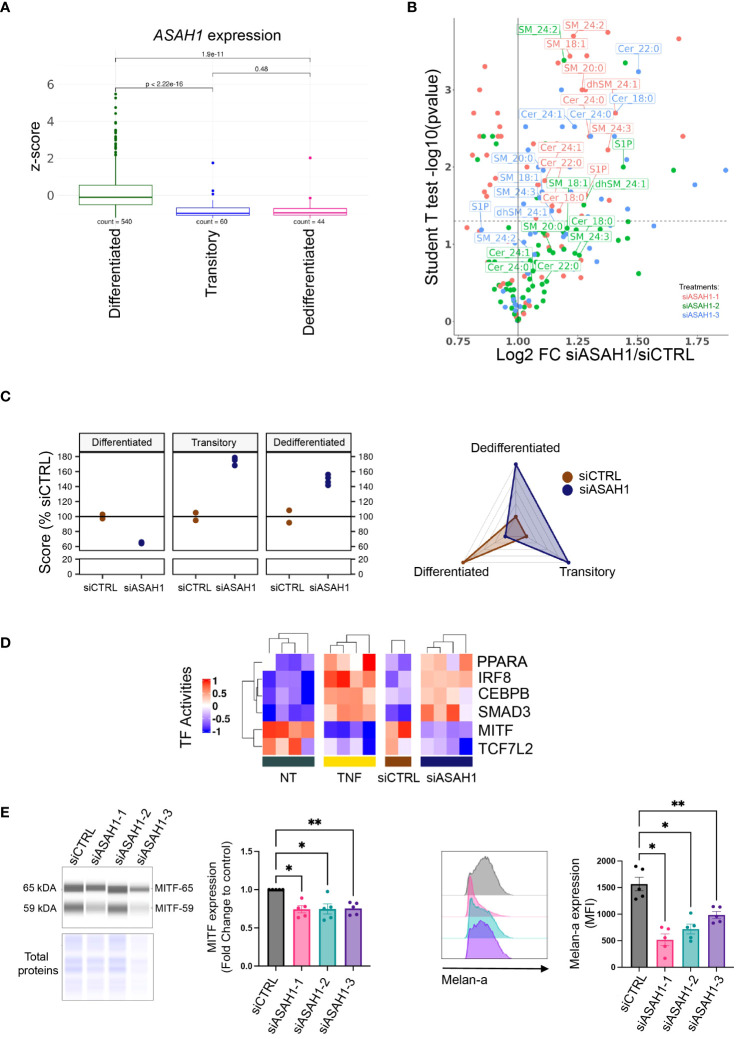
Acid ceramidase silencing leads to melanoma dedifferentiation. **(A)** *ASAH1* gene expression in tumors of melanoma metastatic patients combined from public cohorts (see method) (T test); tumors from TCGA SKCM and Gide/Riaz cohorts were classified in Differentiated/Transitory/Dedifferentiated based on their highest score. **(B)** WM35 melanoma cells were transfected with a control (siCTRL) or 3 siRNAs targeting AC transcript (siASAH1–1, siASAH1–2 or siASAH1–3). Then, the sphingolipid content was analyzed by mass spectrometry 72 hours later. Labeled sphingolipids showed similar changes with at least 2 siRNAs. **(C)** RNA-Seq analysis of WM35 transfected with a siCTRL (n=2) or siASAH1–1 for 72 hours (n=2 in duplicate). Data are represented by dot plots (left) or spider plot (right). **(D)** Heatmap representing the activities of transcription factors that were significantly and similarly modified in WM35 cells during 24 hours incubation with TNF or 72 hours after WM35 transfection with siASAH1–1 (see methods). **(E)** WM35 cells were transfected with 3 siRNAs targeting AC transcript (siASAH1–1, siASAH1–2 or siASAH1–3). 72 hours later, MITF and Melan-a expression was analyzed by simple western and normalized by total proteins (RM One-Way Anova,**p<0.01, *p<0.05, n=5) (left panels) and flow cytometry (RM One-Way Anova, **p<0.01, *p<0.05, n=5) (right panels), respectively.

Among the ceramide metabolism genes being significantly downregulated in melanoma cell lines upon TNF treatment, we identified *ASAH1*, the gene encoding AC, the ultimate enzyme of sphingolipid lysosomal catabolism (**Figure 2B** and **Supplementary Figure 4B**). Subsequently, we validated the impact of TNF on AC specific enzyme activity and protein level. Incubating WM35 melanoma cells with TNF significantly inhibited AC enzyme activity under our experimental conditions (**Supplementary Figure 5A**). In line with this observation, we found that TNF elicited a reduction in AC protein expression as evaluated by Western blot. This reduction extended to both the AC precursor protein and its alpha and beta subunits (**Supplementary Figure 5B**). Notably, prior researches have demonstrated that MITF drives AC expression and that it is downregulated in invasive melanoma cells (20, 37). Accordingly, *ASAH1* expression mirrored the kinetics of melanocytic markers, exhibiting maximum inhibition 24 hours post-TNF treatment (**Supplementary Figure 6A**). Conversely, expression of mesenchymal markers was significantly increased as early as 6 hours after TNF treatment (**Supplementary Figure 6A**). Moreover, the expression of *ASAH1* in tumors from metastatic melanoma patients, combined from public cohorts and classified according to our 3 previously defined scores, is lower in the transitory and dedifferentiation states when compared to the differentiated state (**Figure 3A**).

To investigate whether AC inhibition upon TNF plays a causal role in melanoma cell dedifferentiation, WM35 cells were transfected with siRNA targeting the AC transcript and reducing the AC enzyme activity (**Supplementary Figures 6B, C**). First, the impact of AC knockdown on sphingolipid content was assessed by mass spectrometry. Similar to TNF treatment, AC inhibition led to the accumulation of (dihydro)sphingomyelin and ceramide species (**Figure 3B**). However, only one of the three siRNAs used resulted in monohexosylceramide accumulation. Thus, AC downregulation appears to be partially involved in TNF-induced sphingolipid changes. Moreover, AC knockdown triggered melanoma cell dedifferentiation as evaluated by RNA-Seq analysis (**Figure 3C**). Indeed, the AC knockdown cells exhibited transitory and dedifferentiated phenotypes, indicating that silencing AC alone was sufficient to initiate the dedifferentiation process of melanoma cells. This was further supported by transfecting the WM35 cells with a different siRNA sequence, which promoted the dedifferentiation process as assessed by RT-qPCR (**Supplementary Figure 6D**).

To elucidate the shared molecular mechanisms through which AC knockdown and TNF could instigate melanoma cell dedifferentiation, we conducted a comparative analysis of gene signatures obtained from WM35 melanoma cells treated with TNF for 24 hours (optimum effect) and those subjected to AC knockdown. Remarkably, 28% of total genes modulated by TNF treatment were also affected by AC knockdown (**Supplementary Figure 7A**). These 275 shared genes were associated with various GO biological processes such as “regulation of cell differentiation”, “hallmarks of epithelial-to-mesenchymal transition” and “biological adhesion” (**Supplementary Figure 7A**). Next, we inferred transcription factor (TF) activities on both the TNF-treated and the siASAH1-treated RNA-Seq datasets (**Supplementary Figure 7B**). We focused on TFs commonly regulated between the 24 hours TNF treatment condition and AC knockdown in melanoma cells. This analysis revealed 4 upregulated TFs (PPARα, IRF8, C/EBPβ and SMAD3), and 2 downregulated TFs (MITF and TCF7L2) (**Figure 3D**). In the DoRothEA package, those TFs are supposed to regulate 160 genes (30), among which 9 were commonly regulated by TNF treatment or siASAH1 (*DCT*, *GPR143*, *SNAI2*, *TRPM1*, *LPL*, *NFKBIA*, *PTHLH*, *VEGFA*, *GLCE*). While 6 of those genes (*DCT*, *LPL*, *NFKBIA*, *PTHLH*, *SNAI2*, *VEGFA*) belong to cell differentiation, *GPR143* is part of the pigmentation pathway in the C5 GO pathway database. Accordingly, C/EBPβ, SMAD3 and MITF are directly related to melanoma dedifferentiation (38). Concerning MITF, a positive feedback loop at the gene level between this TF and AC in the context of melanoma phenotype switching has been previously observed (20). Here, we show that transfecting WM35 cells with 3 different siASAH1 reduced MITF protein expression (**Figure 3E**). The impact of AC knockdown on MITF was further confirmed by evaluating the MITF target Melan-a, which expression decreased upon AC silencing (**Figure 3E**).

Collectively, our data indicate that AC silencing, mimics, at least in part, TNF-induced melanoma dedifferentiation, also suggesting that AC is not the only player in this mechanism.

### TNF-induced activation of glucosylceramide synthase also participates in melanoma cell dedifferentiation

**Figure 4:**
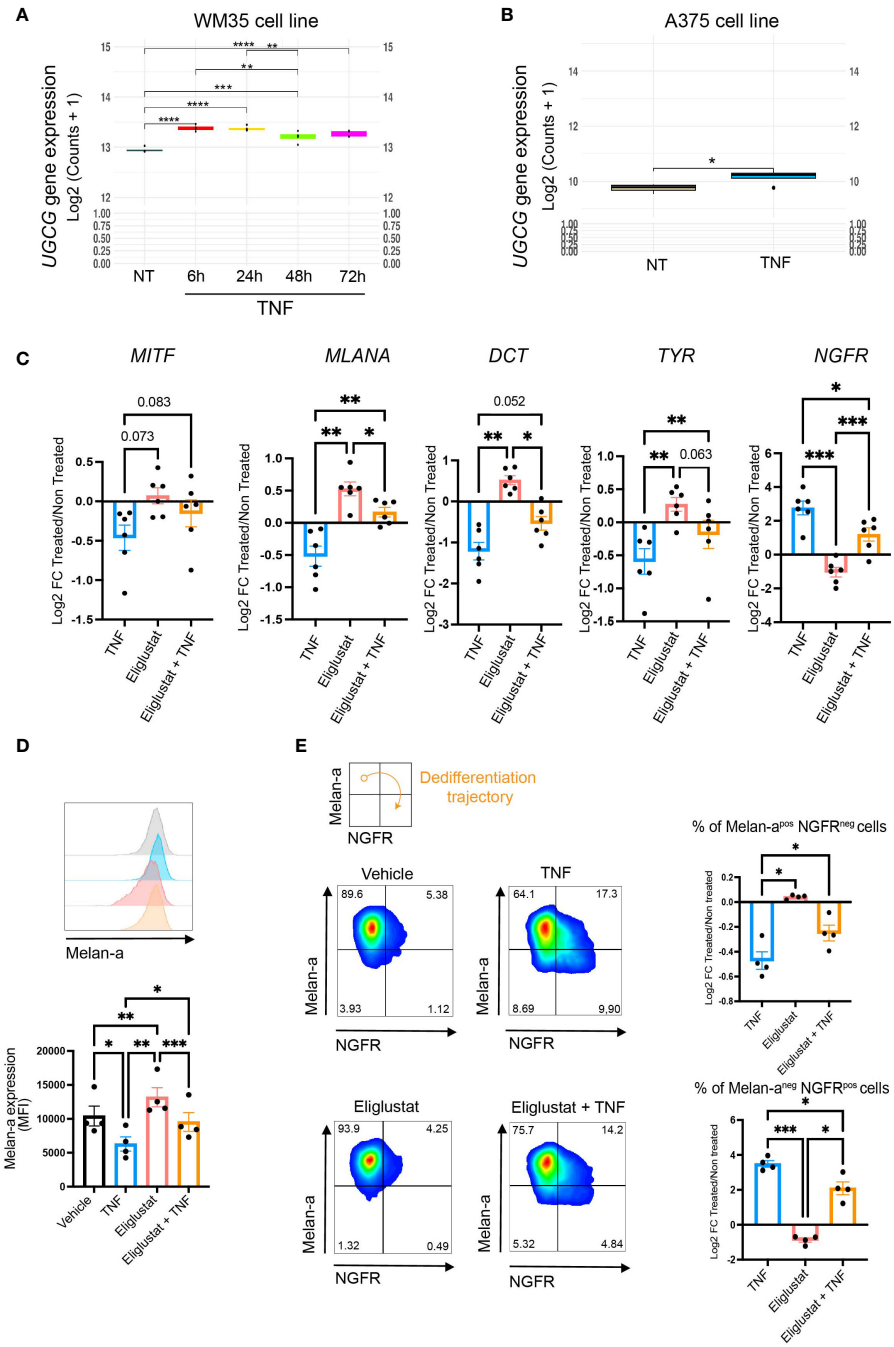
Targeting the glucosylceramide synthase alters TNF-induced dedifferentiation. **(A)** *UGCG* gene expression in WM35 cells treated with 50 ng/mL TNF for 6 (red), 24 (yellow), 48 (green) or 72 hours (pink). **(B)** *UGCG* gene expression in A375 cells treated with 50 ng/ml TNF for 24 hours. A and B, RNAseq data (n=4). **(C)** 451Lu cells were treated with 50 ng/mL TNF for 24 hours in combination, or not, with 6 μM Eliglustat. *MITF*, *MLANA*, *DCT*, *TYR* and *NGFR* gene expression was analyzed by RT-qPCR. Data are means ± sem (RM One-Way Anova, ***p<0.001, **p<0.01, n=6). **(D, E)** 451Lu cells were treated with 50 ng/mL TNF with or without 6 μM eliglustat for 48h as indicated. Melan-a and NGFR protein expression were evaluated by flow cytometry. **(D)** Median fluorescence intensity (MFI) of Melan-a. Inserts: representative images. Data are means ± sem (RM One-Way Anova, ***p<0.001, **p<0.01, *p<0.05, n=5). **(E)** Quantification of Melan-a positive and NGFR negative cells (upper plot) or Melan-a negative and NGFR positive cells (bottom plot). Data are means +/- sem and represented by Log2(Fold Change) compared to control (RM One-Way Anova, *p<0.05, n=5).

As depicted in **Figure 2**, TNF-induced melanoma cell dedifferentiation was also associated with the production of monohexosylceramides and dihexosylceramides. As a matter of fact, TNF increased the expression of glucosylceramide synthase (encoded by the *UGCG* gene) in the WM35, A375 and 451Lu cell lines (**Figures 4A, B** and **Supplementary Figure 4B**). To evaluate the contribution of the glycosphingolipid pathway to the TNF-induced melanoma cell dedifferentiation process, we monitored the impact of eliglustat, an inhibitor of glucosylceramide synthase (GCS), by RT-qPCR on various markers of melanoma dedifferentiation. As expected, TNF decreased the gene expression of *MITF* and its target genes *MLANA*, *DCT* and *TYR*, and, conversely, increased the expression of the stemness factor *NGFR* in 451Lu melanoma cells (**Figure 4C**). Combining TNF with eliglustat partially attenuated the TNF-induced changes (**Figure 4C**). This was confirmed at the protein level, where combining TNF with eliglustat partially reversed TNF-induced decrease of Melan-a (**Figure 4D**). Moreover, although there was no difference in overall NGFR expression (MFI), eliglustat treatment decreased the frequency of the more dedifferentiated population (Melan-a^neg^ NGFR^pos^) that appeared upon TNF treatment (**Figure 4E**). This was further confirmed in the WM35 cell line (**Supplementary Figures 8A, B**).

Overall, our data indicate that TNF triggers the accumulation of ceramide metabolites, including glycosphingolipids, which likely contribute to melanoma cell dedifferentiation.

### Plasma ceramide metabolites predict resistance to immune checkpoint inhibitors in advanced melanoma patients

Despite significant progress in the care of advanced melanoma patients thanks to ICI administration, around half of the patients do not respond to this immunotherapy. So far, the best long-term therapeutic response can be achieved with the ipilimumab and nivolumab combination (1). Identification of resistance biomarkers to ICI is urgently needed to better stratify patients for therapy. As changes in sphingolipid metabolism contribute to TNF-induced melanoma dedifferentiation, we hypothesized that some circulating sphingolipids may constitute biomarkers to predict resistance to immunotherapy.

**Figure 5:**
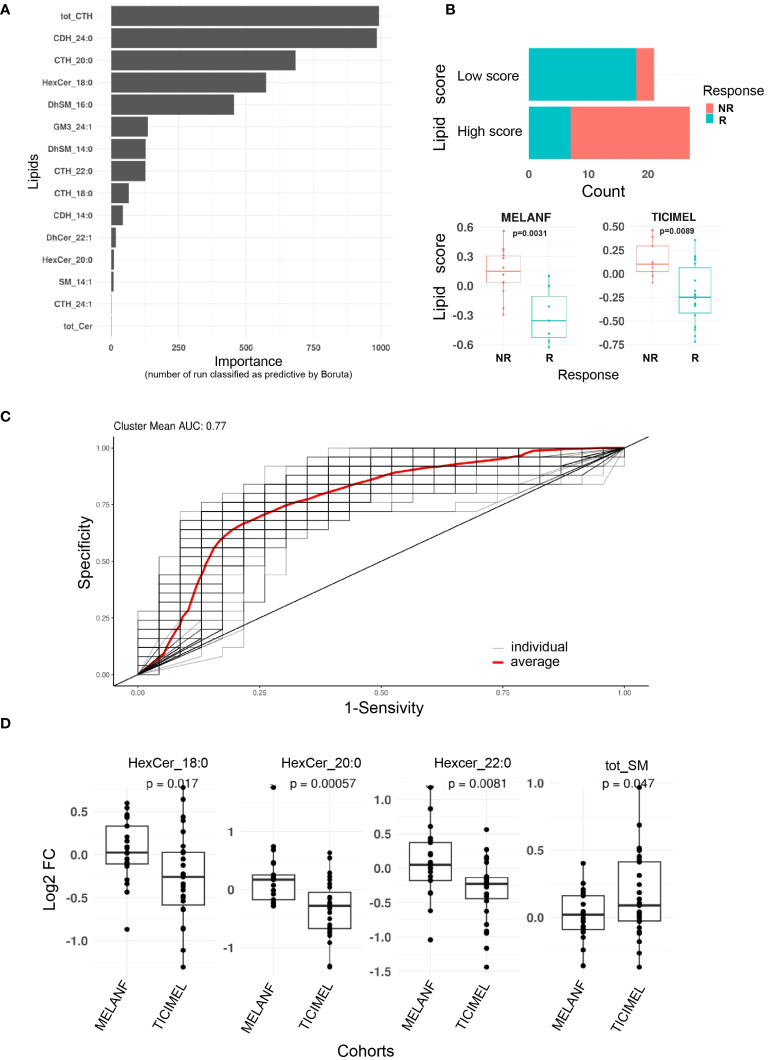
Plasma ceramide metabolites predict resistance to immune checkpoint inhibitors in advanced melanoma patients. Ceramide metabolite plasma levels were measured in advanced melanoma patients treated with ipilimumab (anti-CTLA-4) and nivolumab (anti-PD-1) in combination (patients from TICIMEL clinical trial) or not (patients from MELANFα clinical trial) with anti-TNF at baseline and week 6 post-treatment induction [n=48 paired pre/post treatment including 25 responders (R, including patients with complete and partial responses) and 23 progressors (NR, including non-responder patients and patients with stable disease) from patients enrolled in TICIMEL or MELANFα clinical trials]. **(A)** For each ceramide metabolite, the evolution between week 6 post-treatment induction and baseline, was calculated as Log2(Fold Change). Boruta algorithm was then applied 1,000 times to identify which evolution of ceramide metabolite levels predicted the resistance of melanoma patients to immunotherapy (n refers to the number of times the features were classified as predictive by Boruta). **(B)** A score from the top eight ceramide metabolites as identified in A was established [mean (Fold changes of lipids being higher in non-responders)-mean (Fold changes of lipids being lower in non-responders)]. Scores above 0 and below 0 were considered as high and low, respectively. Score enrichment between responders and non-responders was significantly different (Chi2 test, p=0.00013). The evolution of this score in responders and non-responders from MELANFα and TICIMEL is shown in the lower panels (Wilcoxon test). **(C)** A receiver operating characteristic curve (ROC) were performed (binomial, stratified 5 k-fold, 100 times) from the top eight predictive features with an AUC=0.77 (red curve). **(D)** comparison of ceramide metabolite evolution between patients from TICIMEL (n=27 pre-treatment and post-treatment) and MELANFα (n=21 pre-treatment and post-treatment) clinical trials (t test).

We leveraged data from the clinical trials MELANFα (NCT03348891) and TICIMEL (NCT03293784), involving 48 advanced melanoma patients treated with ICI or ICI in combination with anti-TNF (paired pre- and post-treatment samples). To evaluate whether plasma levels of ceramide metabolites could predict clinical outcomes at week 12, we quantified ceramide metabolites in patients’ plasma by mass spectrometry at baseline and at week 6 post-treatment induction. Then, we constructed a predictive model and employed the Boruta algorithm to evaluate feature of importance and determine the top sphingolipids whose evolution along therapy proved to be the most reliable predictors. Among 78 sphingolipids, the increase of the total amount of tri-hexosylceramides (CTH) emerged as the most predictive feature (**Figure 5A**). The next 3 best predictive features were also associated with glycosphingolipid metabolism and consisted of the increase of C24:0 di-hexosylceramide, C20:0 trihexosylceramide and C18:0 monohexosylceramide (**Figure 5A**). Using the top 8 best predictive sphingolipids, we created a sphingolipid score predictive of the response to ICI. In our cohorts of patients, a low sphingolipid score was significantly associated with a better response to ICI (**Figures 5B**) and achieved an area under the ROC curve of 77% (**Figure 5C**).

Furthermore, to evaluate the impact of TNF-dependent signaling on the evolution of the plasma sphingolipidome between baseline and week 6, we compared the sphingolipid patterns in the plasma of advanced melanoma patients treated with ipilimumab and nivolumab (i.e., bi-therapy) from the MELANFα (NCT03348891) clinical trial, with those measured in the TICIMEL clinical trial (NCT03293784), in which patients were co-administered with ipilimumab, nivolumab and anti-TNF (certolizumab or infliximab) (i.e., tri-therapy). When co-administered with ipilimumab and nivolumab, anti-TNF decreased the monohexosylceramide plasma content, with significant differences for C18:0, C20:0 and C22:0 monohexosylceramides and increased the total sphingomyelin plasma content (**Figure 5D**).

Collectively, our data indicate that, in the context of ipilimumab and nivolumab therapy, the glycosphingolipid pattern in plasma may predict the clinical outcome of advanced melanoma patients treated with ipilimumab and nivolumab. Moreover, the TNF-dependent signaling pathway contributes to producing circulating monohexosylceramides, which are precursors for more complex glycosphingolipids putatively involved in resistance to ICI.

## Discussion

The present study shows that TNF-induced changes in ceramide metabolism contribute to melanoma cell dedifferentiation, a process which facilitates melanoma progression and resistance to immunotherapies (9). Among the metabolic pathways involved, we identified the inhibition of the acid ceramidase (AC) as well as the *de novo* sphingolipid synthesis pathway as being triggered by TNF.

The role of AC in melanoma has been widely investigated. On the one hand, AC is likely involved in melanoma cell proliferation and tumor growth *in vivo*. First, AC is highly expressed in proliferative melanoma cell lines (20, 39). Second, AC knockdown in melanoma cells reduces tumor growth *in vivo* (40). Finally, a complete AC ablation by a CRISPR/Cas9 technique in the A375 melanoma cell line leads to ceramide accumulation, cell cycle arrest in G1/S, senescence and apoptosis (37, 41). Thus, AC seems to be crucial for melanoma cell survival and proliferation. On the other hand, AC expression is lower in invasive cells than in proliferating cells. On tumor biopsy sections, its expression is greater at the level of the superficial sites than in the invasive zones (20). Reducing AC expression by RNA interference increases melanoma cells’ migration capacity. Thus, low AC expression is associated with a more invasive phenotype in melanoma cells (20).

To the best of our knowledge, whereas AC has been reported to modulate proinflammatory and cytotoxic TNF signaling pathways in different cell types (16, 42, 43), the present study is the first showing that TNF-induced AC inhibition contributes to the melanoma cell dedifferentiation process. The mechanism by which TNF decreases AC expression likely involves the inhibition of MITF, which has been identified to target the promoter of AC’s cognate gene *ASAH1* (20). AC inhibition may contribute to maintaining the dedifferentiation phenotype of melanoma cells, by enhancing the accumulation of ceramide as previously shown for the acid sphingomyelinase (44). Moreover, AC knockdown in WM35 cells was sufficient to enhance the activities of C/EBPβ and SMAD3. Our findings are in line with studies indicating that C/EBPβ, which is activated by exogenous ceramides (45, 46), represses MITF transcription in melanoma (38). Moreover, endogenous ceramides were shown to stimulate the TGFβ/SMAD3 signaling pathway (47). Thus, TNF-induced changes in ceramide metabolism likely contribute to melanoma dedifferentiation and aggressiveness.

The alterations of ceramide metabolism upon TNF treatment in melanoma cells do not seem to be restricted to the AC inhibition. Indeed, TNF elicited the increase of *de novo* sphingolipid synthesis, leading to the accumulation of dihydroceramides, ceramides, dihydrosphingomyelins, sphingomyelins, monohexosylceramides and dihexosylceramides. However, as we noticed only the accumulation of (dihydro)sphingomyelins and ceramide species in AC knockdown cells, the inhibition of AC does not entirely recapitulate TNF-induced ceramide metabolism changes. Incubation of melanoma cells with subtoxic ceramide concentrations triggered cell dedifferentiation. Conversely, blocking *de novo* ceramide synthesis impaired TNF-induced melanoma cell dedifferentiation. This implies that ceramides or ceramide metabolites cause melanoma cell dedifferentiation. The role of ceramide metabolites in TNF-induced melanoma cell dedifferentiation is further documented by the inhibition of this process by eliglustat, pointing to the role of glycosphingolipids in melanoma cell dedifferentiation upon TNF treatment. Thus, GCS may represent a valuable target in melanoma to prevent TNF-induced immune escape and progression. Accordingly, targeting GCS by epigenetic or pharmacological approaches reduced the glucosylceramide and ganglioside content of MEB4 mouse melanoma cells without affecting cell proliferation and viability *in vitro* but greatly impaired the tumor progression in immunocompetent mice (48). Whereas the impact of GCS inhibition on the immune response remains to be evaluated, it is tempting to speculate that targeting the GCS could prevent melanoma cell dedifferentiation, contributing to enhancing immune response against melanoma cells.

Herein, we provide evidence that alterations in sphingolipid metabolism after treatment induction may be putative biomarkers to predict ICI resistance. We identified a sphingolipid score mainly composed of glycosphingolipid metabolites whose increased content in the plasma of melanoma patients treated with ipilimumab and nivolumab is associated with poor response to treatment. This further indicates that alterations in ceramide metabolism may contribute to resistance to immunotherapy targeting immune checkpoints such as PD-1 and CTLA-4. Whereas plasma glycosphingolipids do not necessarily derive from the tumors, part of them might be released by melanoma cells, since various melanoma cell lines contained glycosphingolipids at high levels (49). Moreover, comparing the evolution of the plasma sphingolipidome between patients enrolled in the TICIMEL and MELANFα clinical trials showed that anti-TNF reduced the production of monohexosylceramides in plasma, further supporting that TNF-dependent signaling contributes to glycosphingolipid accumulation in the context of ICI therapy in advanced melanoma patients. The production of lactosylceramide by melanoma cells was shown to favor melanoma aggressiveness. For instance, the lactosylceramide synthase β4GalT5 of B16F10 mouse melanoma cells facilitates melanoma progression in mice (50). Moreover, a study showed that human melanoma cells with resistance to Braf inhibitors underwent intense metabolism rewiring, including accumulation of hexosylceramides (51). Whether or not plasma sphingolipids are relevant biomarkers for patients treated with ICI will be further evaluated in the context of the Immusphinx clinical trial (NCT03627026), the primary objective of which is to determine sphingolipid signatures predicting resistance/response to ICI in advanced melanoma patients.

Altogether, our study highlights for the first time that ceramide metabolism alterations contribute to TNF-induced melanoma cell dedifferentiation and opens new avenues for developing original therapeutic strategies aiming at reducing melanoma progression and resistance to therapies. Moreover, we identified a plasma sphingolipid score, the increase of which predicts the resistance to immune checkpoint inhibitors in advanced melanoma patients.

## Data availability statement

RNA seq data obtained in WM35 (GSE270741) and A375 (GSE270740) melanoma cells are deposited in Gene Expression Omnibus (GEO) repository.

## Ethics statement

The MELANFα trial protocol was reviewed and approved by the French committee for the protection of persons and the French drug agency (Agence Nationale de Sécurité du Médicament et des produits de santé; date of approval January 24, 2018; EudraCT 2017-A02511-52). MELANFα is registered under ClinicalTrials.gov, number NCT03348891. The TICIMEL trial protocol was reviewed and approved by the French committee for the protection of persons and the French drug agency (Agence Nationale de Sécurité du Médicament; date of approval August 4, 2017 EUDRACT 2016-005139-34). All study procedures were carried out in accordance to the International Council for Harmonization tripartite guideline on good clinical practice (Helsinki declaration). An Independent Data Monitoring committee (IDMC) monitored and evaluated data from the TICIMEL study. The TICIMEL study is registered under ClinicalTrials.gov, number NCT03293784. The informed consent to be enrolled in either trial was obtained for each patient.

## Author contributions

CD: Conceptualization, Data curation, Formal analysis, Investigation, Methodology, Visualization, Writing – original draft, Writing – review & editing. MG: Data curation, Formal analysis, Investigation, Methodology, Visualization, Writing – original draft, Writing – review & editing. EM: Investigation, Writing – review & editing. BJ: Investigation, Writing – review & editing. VG: Investigation, Writing – review & editing. AM: Investigation, Methodology, Writing – review & editing. MT: Formal analysis, Visualization, Writing – review & editing. CC: Resources, Writing – review & editing. JM: Resources, Writing – review & editing. TL: Writing – review & editing. J-PD: Resources, Writing – review & editing. NM: Resources, Writing – review & editing. VP: Supervision, Validation, Writing – review & editing. NA-A: Conceptualization, Formal analysis, Funding acquisition, Supervision, Project administration, Writing – original draft, Writing – review & editing. BS: Conceptualization, Formal analysis, Funding acquisition, Supervision, Project administration, Writing – original draft, Writing – review & editing.
