# Supplementary material for: Ceramide metabolism alterations contribute to Tumor Necrosis Factor-induced melanoma dedifferentiation and predict resistance to immune checkpoint inhibitors in advanced melanoma patients
Source: Front Immunol. 2024 Jul 29;15:1421432. doi: 10.3389/fimmu.2024.1421432 (PMC11317267; doi:10.3389/fimmu.2024.1421432)
Supplement: Supplementary file 1 [file DataSheet_1.docx]

Supplementary Material

**Supplementary Materials and Methods**

**RNA-Seq on public data:** three public RNA-Seq datasets were retrieved for the present paper with expression datasets in pre-treatment samples from anti-PD-1-treated patients. Stable disease patients with RECIST criteria and bitherapy (anti-PD1+anti-CTLA4) were filtered out. The 3 datasets are Gide *et al.* (1), composed of 19 responders and 16 progressors, Riaz *et al.* (2), composed of 10 responders and 23 progressors, and Liu *et al.* (3), composed of 47 responders and 58 non responders/progressors, for a total of 173 samples. Batch correction was performed using Combat-Seq function, part of the sva package (4), and normalization was done with the DESEQ2 [[10.18129/B9.bioc.DESeq2](https://doi.org/doi:10.18129/B9.bioc.DESeq2)] pipeline.

**Definition of differentiation state signatures from RNA-Seq data:** Cell differentiation state gene signatures were recovered from literature, including Hoek_PRO/Hoek_INV signatures using microarray gene expression datasets on 86 melanoma cultures (5), signatures from human melanoma cell lines and patient tumors (6), Woodman_MEL (differentiated state)/Woodman_NPLAS (transitory state)/Woodman_MES (dedifferentiated state) signatures (7), Wouters signatures from single cell and bulk RNAseq from melanoma cultures (8), Verfaillie et al from patient biopsies and melanoma cultures (9), and finally from PDX in rambow et al (10).

**RT-qPCR analyses:** After treatment, the cells were washed with PBS, detached by trypsin and centrifuged (1,500 rpm, 3 min). RNA was then extracted according to the supplier's protocol (RNeasy kit, Qiagen). RNA concentration was determined using a Clariostar. One μg of RNA was reverse transcribed using the RT iScript enzyme (Biorad). 25 ng of cDNA was used as template for quantitative PCR. Reactions were performed in the StepOne thermal cycler (Applied Biosystems) using the QuantiFast SYBR Green Master Mix and the probes of interest. Results were quantified using the StepOne system software. The cDNAs of mRNAs encoding GAPDH and HPRT were used to normalize human gene expression. The rate of change of stimulated target gene expression compared to the unstimulated control was determined according to the following formula: rate of change = 2-ΔΔCt, where ΔΔCt = (Target Ct - Control Ct) stimulated - (Target Ct - Control Ct) unstimulated. The Ct values were defined as the cycle number in which the fluorescence signals are detected.

**Flow cytometry analyses:** After treatment, the cells were detached by trypsin, washed with PBS, then centrifuged (1,500 rpm, 3 min.). The cell pellets were then suspended in 100 µL of FACS buffer (PBS 0.5% BSA, 2 mM EDTA) and placed in 96-well V bottom plates. The cells were then incubated for 30 min in the dark in FACS buffer containing a mix of antibodies targeting membrane markers and a viability marker (Live/Dead, Thermofisher #L3224). At the end of the incubation, cells were centrifuged and washed twice with FACS buffer, then incubated for 30 min in fixation/permeabilization buffer (eBioscience, #00-5223-56, #00-5123-43). After washing with permeabilization buffer, cells were incubated with antibodies targeting intracellular markers.  After 2 washes, the cells were analyzed by flow cytometry (LSRII BD). The following antibodies were used at the indicated dilutions: AF647-coupled anti-NGFR (1/200; BD Pharmingen, C40-1457); PE-Cy7-coupled anti-AXL (1/200; eBiosience, 25-1087-42); PE-coupled anti-Melan-a (1/200; Santa Cruz Biotechnology, sc-20032-PE).

**Simple Western analyses:** After treatment, the cells were detached by trypsin, washed with PBS, then centrifuged (1500 rpm, 3 min.). The cell pellets were suspended in 100 µL of RIPA buffer (RIPA buffer; Sigma #R0278) and sonicated for 20 seconds, 40% amplitude and then centrifuged for 10 minutes at 14,000 g at 4°C. The supernatants were collected and used for protein quantification by BCA assay and then diluted in the fluorescent mastermix and dispensed into the plate with all the reagents of the Simple Western kit (Separation module 12-230 kDa; Protein Simple #SM W004). The results were analyzed using the Compass for Simple Western software.

**Western blot analyses:** After treatment, proteins were extracted in cell lysis buffer (Cell Signaling Technology) and 30 μg of proteins were electrophoresed on 7.5% to 12.5% SDS-PAGE, transferred to a nitrocellulose membrane (Perkin-Elmer) and labelled with a rabbit polyclonal anti-acid ceramidase antibody (a kind gift from Dr. K. Sandhoff, Bonn, Germany) or monoclonal anti-β-Actin (Clone 13E5) (Cell Signaling Technology). Proteins were detected with Chemidoc using an ECL detection system (Bio-Rad) and quantified using ImageJ.

**Acid ceramidase activity:** Acid ceramidase activity was measured as previously reported (11). Briefly, cells were collected and washed twice with PBS. The cell pellets were suspended in 100 µL of 0.2 M sucrose solution and sonicated. The cell homogenates were centrifuged at 15,000 *g* for 3 min. The supernatant was collected and used for protein quantification by BCA assay. The enzyme assay was performed in 96-well plates. Briefly, each well contained a mixture of 74.5 µL of 25 mM sodium acetate buffer pH 4.5, 0.5 µL of a 4 mM Rbm14-12 substrate solution in ethanol (final substrate concentration 20 µM; final ethanol concentration 0.5%), and a fixed amount of protein (10-25 µg) in a 25 µL volume of 0.2 M sucrose solution. The negative control samples were made up of the same incubation mixture in the absence of protein extracts. The plate was incubated at 37°C for 3 h without shaking. Then, the enzyme reaction was stopped by adding 50 µL of methanol and 100 µL of a fresh solution of 2.5 mg/mL NaIO4 in 100 mM glycine/NaOH buffer pH 10.6 to each well. The plate was protected from light for 2 h and then the released fluorescence was quantified using a microplate fluorescence reader (λex 360 nm, λem 446 nm). The amount of umbelliferone released was calculated from the fluorescence intensity using calibration curves with umbelliferone in the range of 0-3,000 pmol.

**Supplementary figures**

**
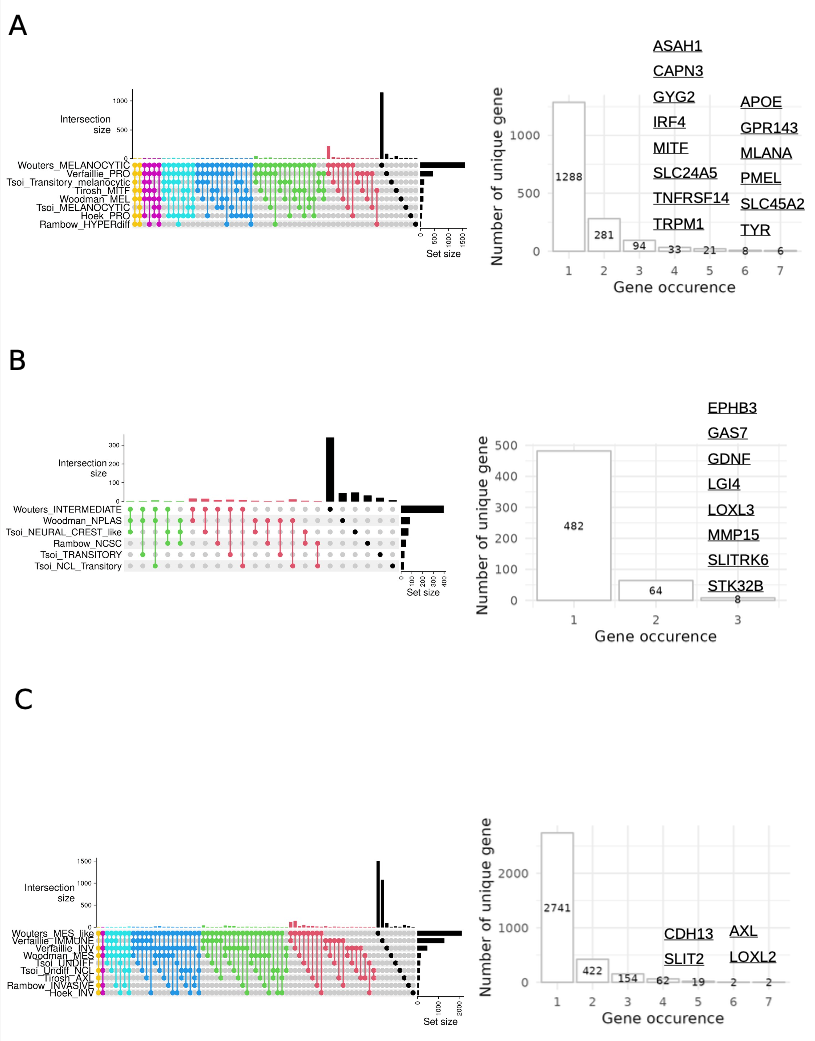
**

**Supplementary Figure 1: Gene occurrence in signatures within the 3 differentiation clusters in proportional Euler plots and its related occurrence barplots**. **A,** Upset plot representing overlaps of genes and occurrence barplot of signature in the differentiated cluster (Genes most present n=6 and 7 are shown). **B,** Upset plot representing overlaps of genes and occurrence barplot of signature in the transitory cluster (Genes most present n=3). **C,** Upset plot representing overlaps of genes and occurrence barplot of signature in the dedifferentiated cluster (Genes most present n=6 and 7).


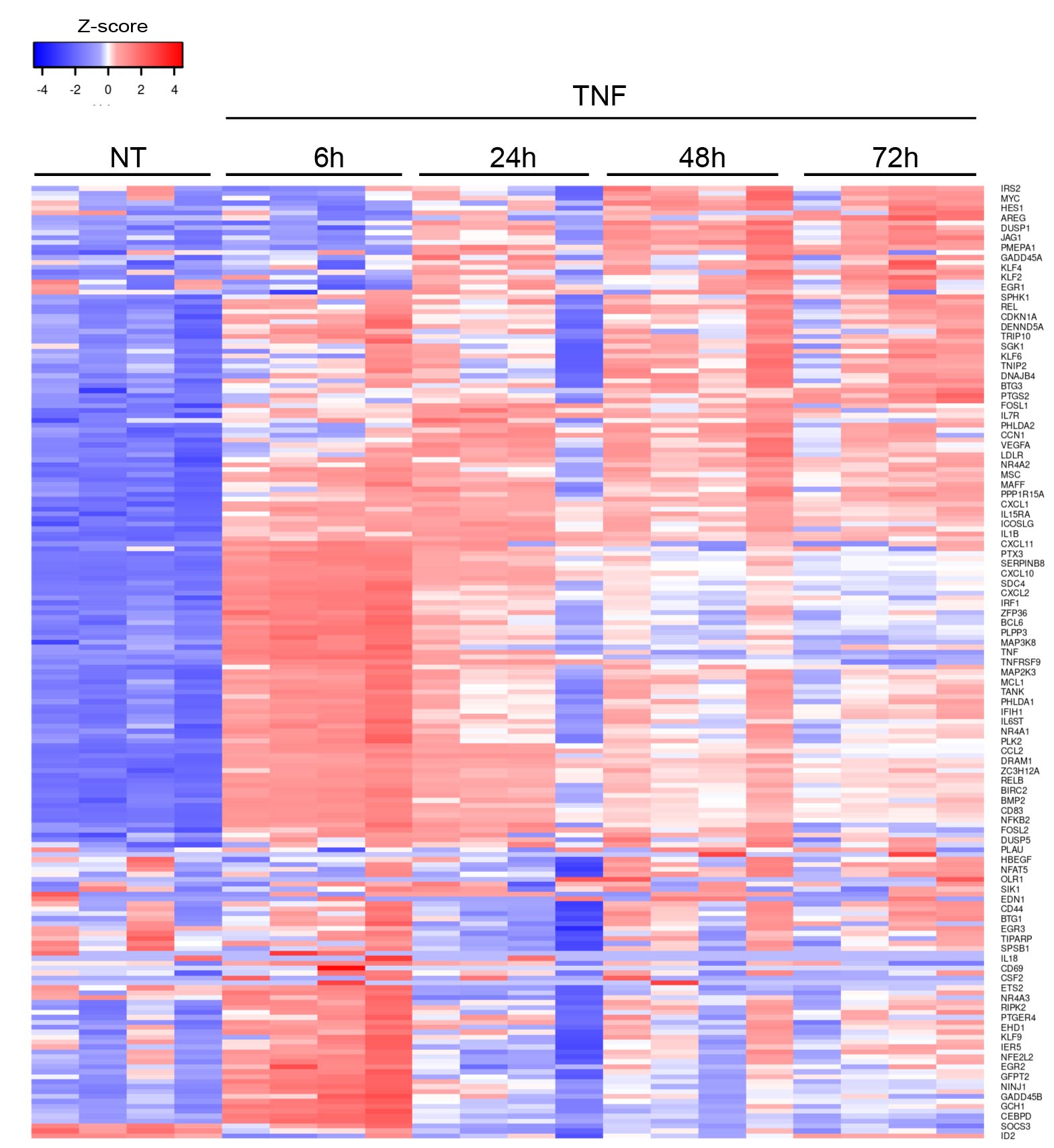


**Supplementary Figure 2: Expression of genes belonging to “Hallmark TNF signaling via NFKB” in TNF-treated and non-treated WM35 melanoma cells.** WM35 melanoma cells were treated or not with TNF as described in the legend to Figure 1B. Heatmap showing the gene expression (RNAseq, n=4).

**
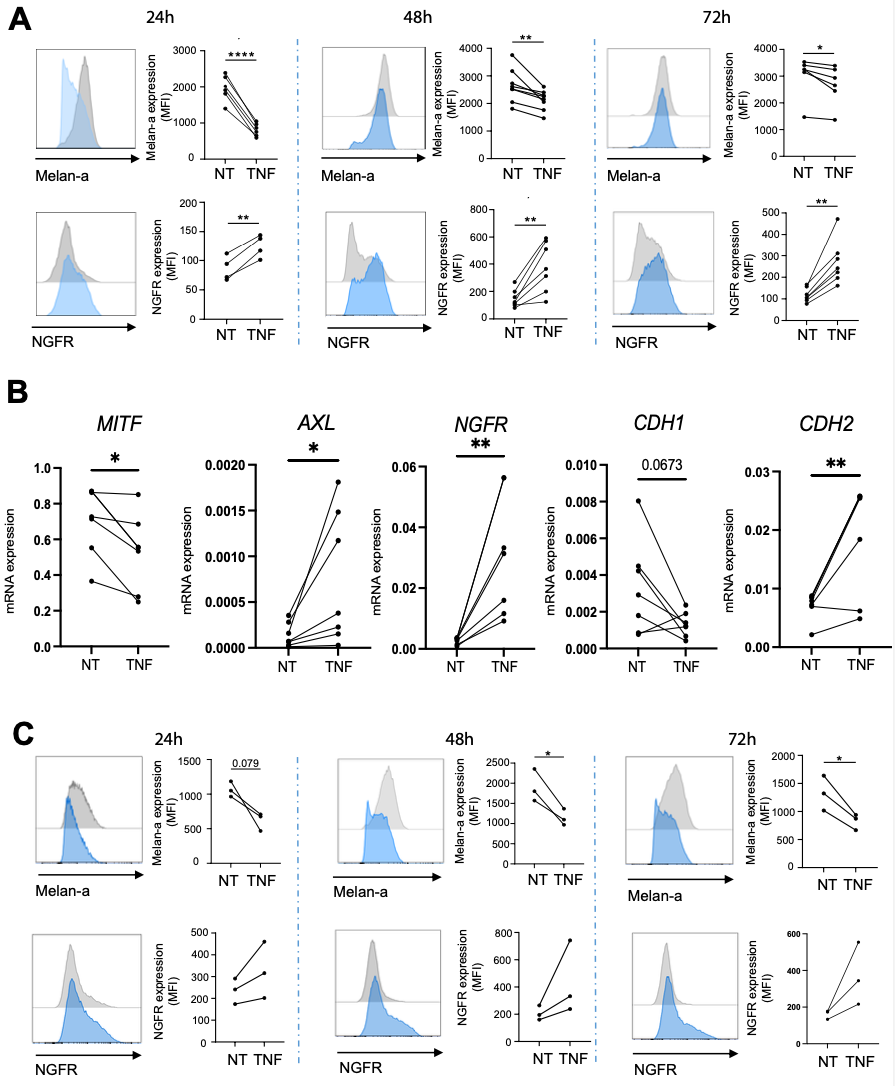
**

**Supplementary Figure 3: Impact of TNF signaling on melanoma cell dedifferentiation and sphingolipid metabolism gene expression. A and C,** WM35 **(A)** or 451Lu **(C)** were treated with 50 ng/mL TNF for 24, 48 or 72 hours. Melan-a and NGFR expression were evaluated by flow cytometry. MFI: Median Fluorescence Intensity. (Paired T Test, * p<0.05, ** p<0.01, **** p<0.0001, n=3-7). **B,** 451Lu cells were incubated with 50 ng/mL TNF for 72 hours and mesenchymal markers (NGFR, AXL, N-Cadherin) and melanocytic markers (MITF, E-Cadherin) gene expression was evaluated by RT-qPCR. (Paired T Test, * p<0.05, ** p<0.01, n=6).

**
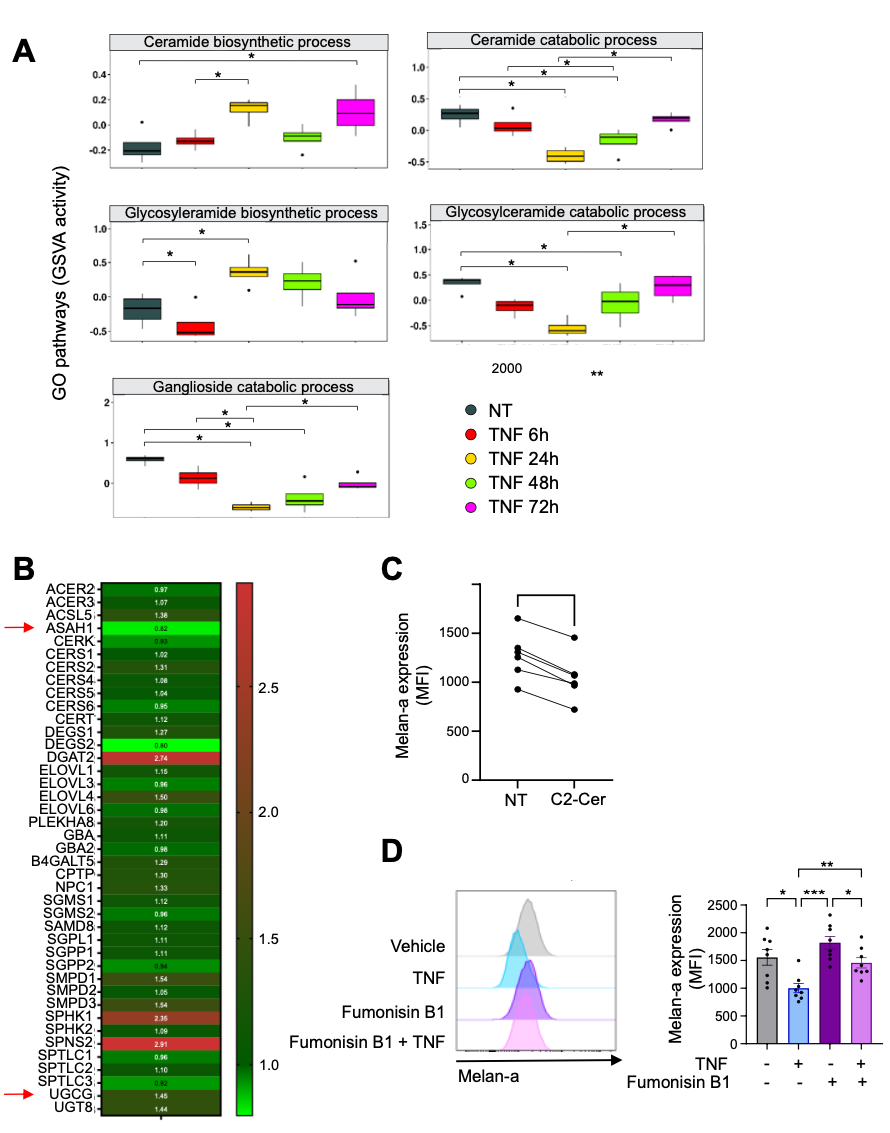
**

**Supplementary Figure 4: TNF-induced ceramide synthesis contributes to melanoma cell dedifferentiation. A,** WM35 melanoma cells were treated with 50 ng/mL TNF for 6, 24, 48 or 72 hours, and then analyzed by RNA-Seq (n=4). Pathway activities inferred by GSVA (see Methods) of the sphingolipid-related biological processes (GO) along TNF treatment (Wilcoxon, *p<0.05). **B,** PCR Array targeting sphingolipid metabolism genes on 451Lu treated with 50 ng/mL TNF for 72 hours. Data are represented by Fold Change compared to control (n=2). The arrows indicate the *ASHA1* and *UGCG* relative gene expression. **C,** WM35 cells were treated with exogenous C2-ceramide (10 µM) for 24 hours and Melan-a expression was evaluated by flow cytometry (Paired T Test, ** p<0.01, n=6). **D,** WM35 cells were treated with 50 ng/mL TNF for 48 hours in combination, or not, with the ceramide synthase inhibitor Fumonisin B1 (5µM). Melan-a expression was evaluated by flow cytometry. Inserts: representative images. MFI: Median Fluorescence Intensity. Data are means ± sem (Paired T Test, * p<0.05, ** p<0.01, *** p<0.001, n=8).

**
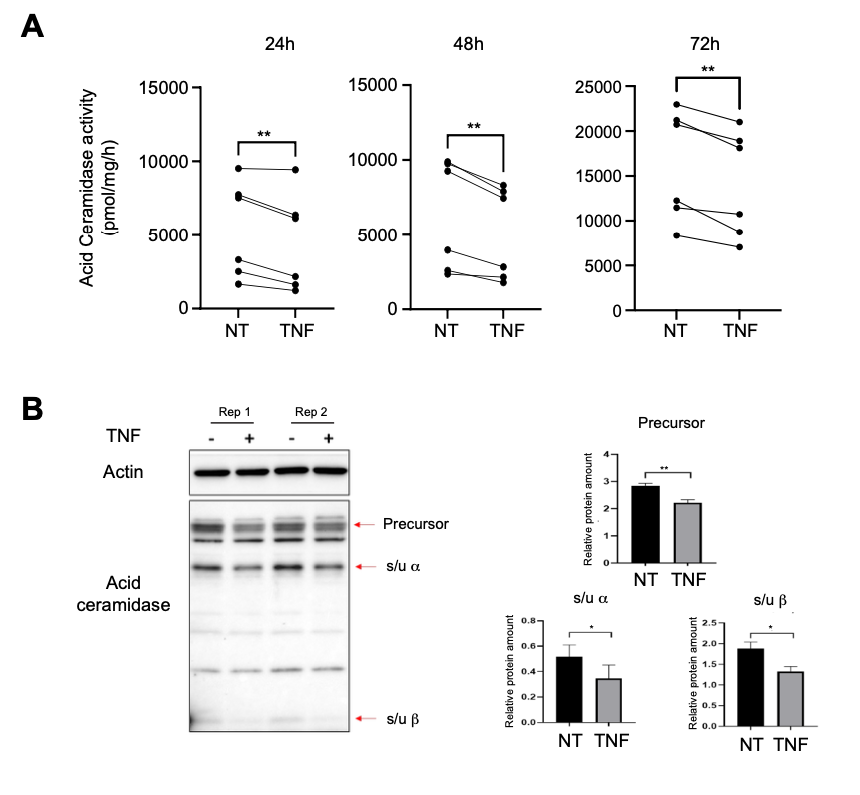
**

**Supplementary Figure 5: TNF decreases acid ceramidase expression and activity. A,** Enzymatic assay of acid ceramidase activity in WM35 melanoma cells treated with 50 ng/mL TNF during 24, 48 or 72 hours. Data are means ± sem (Paired T Test, * p<0.05, ** p<0.01, n=6). **B,** Acid ceramidase protein expression in WM35 melanoma cells treated with 50 ng/mL of TNF during 72 hours. Representative Western Blot of 3 independent experiments in duplicate. (Student t Test, **p<0.01, *p<0.05, n=3).

**
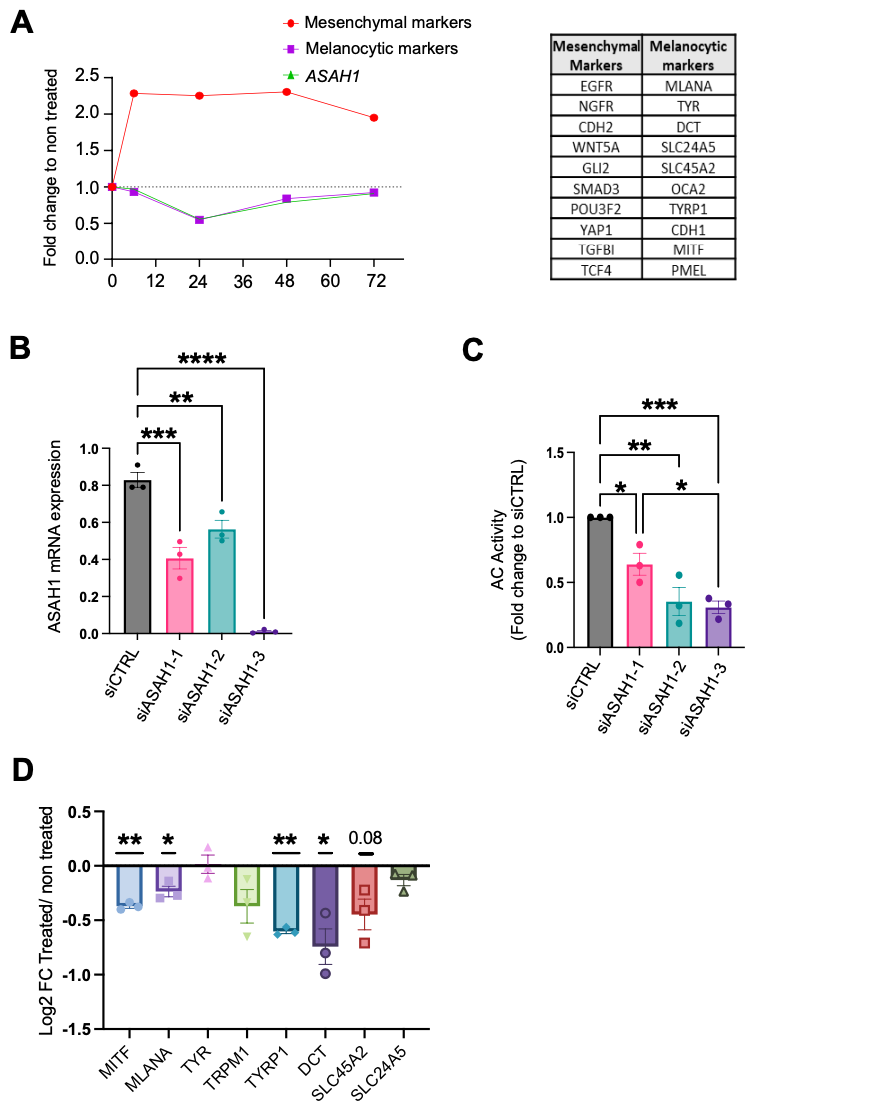
**

**Supplementary Figure 6: Expression of acid ceramidase and melanocytic markers is tightly associated in WM35 melanoma cells. A,** Gene expression profile of *ASAH1* (green curve), mesenchymal markers (red curve) and melanocytic markers (purple curve) in WM35 melanoma cells upon TNF treatment (6, 24, 48, or 72 hours). Data are represented by the fold change compared to the untreated cells. Mesenchymal and melanocytic marker data represent the mean expression of the gene list presented in the table. **B,** WM35 cells were transfected with 3 different siASAH1 for 48 hours and ASAH1 gene expression was evaluated by RT-qPCR. Data are means ± sem. (One-way Anova, ** p<0.01, *** p<0.001, **** p<0.0001, n=3). **C,** Enzymatic assay of acid ceramidase activity in WM35 melanoma cells transfected with 3 different siASAH1 for 48 hours. Data are means +/- sem and are represented by the fold change compared to control (One-way Anova, * p<0.05, ** p<0.01, *** p<0.001, n=3). **D,** WM35 cells were transfected with siASAH1-3 and MITF and its target gene expression was evaluated by RT-qPCR. Data are from 3 independent experiments represented by log2(Fold Change) compared to control (One sample T test, * p<0.05, ** p<0.01, n=3).

**
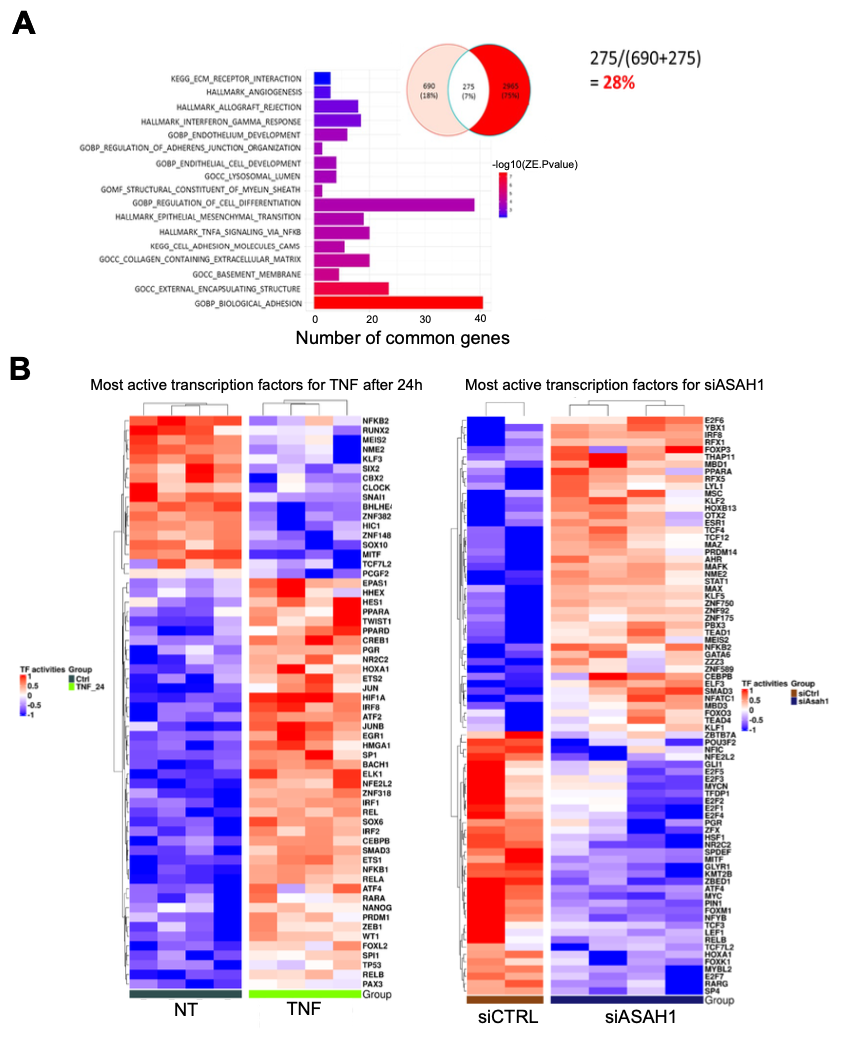
**

**Supplementary Figure 7: TNF treatment and acid ceramidase downregulation share a common invasive dedifferentiation program. A,** Visualization of the signaling pathways significantly involved by the common differentially expressed genes between the TNF-treated WM35 cells at 24 hours and the siASAH1-transfected WM35 melanoma cells at 72 hours. For each pathway, the bar of the histogram indicates the number of common genes differentially expressed belonging to the described pathway. The color of the bar represents the p-value (Zelen exact test); from blue (least significant) to red (most significant). Venn diagram illustrates the intersection between the significantly different gene list between untreated and TNF-treated cells at 24 hours (left) and that between transfected cells with siCTRL and siASAH1 (right). 28% of gene expression being modulated by TNF was commonly modulated upon AC knockdown (pvalue=6.11e-27). **B,** Heatmap representing significant activities of transcription factors in TNF-treated WM35 melanoma cells (TNF) vs untreated cells (NT) at 24 hours (left) and AC knockdown WM35 melanoma cells (siASAH1) vs control (siCTRL) at 72 hours (right).

**
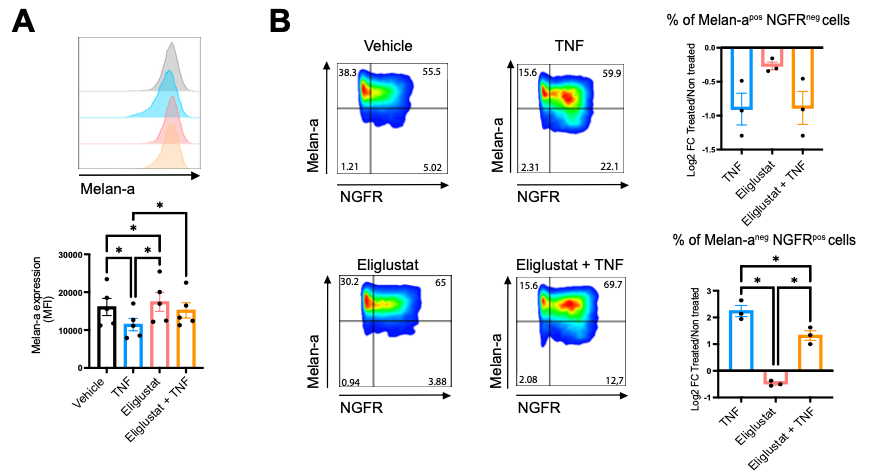
**

**Supplementary Figure 8: Targeting the glucosylceramide synthase alters TNF-induced dedifferentiation in WM35 cells. A and B,** WM35 cells were treated with 50 ng/mL TNF with or without 6 μM eliglustat for 48h as indicated. Melan-a and NGFR protein expression were evaluated by flow cytometry. (D) Median fluorescence intensity (MFI) of Melan-a. Data are means ± sem (***p<0.001, **p<0.01, *p<0.05, RM One-Way Anova, n=5). (E) Quantification of Melan-a positive and NGFR negative cells (upper plot) or Melan-a negative and NGFR positive cells (bottom plot). Data are means +/- sem and represented by Log2(Fold Change) compared to control (*p<0.05, RM One-Way Anova, n=3).

**Supplementary references**

1. Gide TN, Quek C, Menzies AM, Tasker AT, Shang P, Holst J, et al. Distinct Immune Cell Populations Define Response to Anti-PD-1 Monotherapy and Anti-PD-1/Anti-CTLA-4 Combined Therapy. Cancer Cell. 2019;35(2):238-55 e6.

2. Riaz N, Havel JJ, Makarov V, Desrichard A, Urba WJ, Sims JS, et al. Tumor and Microenvironment Evolution during Immunotherapy with Nivolumab. Cell. 2017;171(4):934-49 e16.

3. Liu D, Schilling B, Liu D, Sucker A, Livingstone E, Jerby-Arnon L, et al. Integrative molecular and clinical modeling of clinical outcomes to PD1 blockade in patients with metastatic melanoma. Nat Med. 2019;25(12):1916-27.

4. Leek JTJ, W. E.; Parker, H. S.; Fertig, E. J.; Jaffe, A. E.; Zhang, Y.; Storey, J. D.; Collado Torres, L. sva: Surrogate Variable Analysis. R package version 3.42.0. 2021.

5. Hoek KS, Schlegel NC, Brafford P, Sucker A, Ugurel S, Kumar R, et al. Metastatic potential of melanomas defined by specific gene expression profiles with no BRAF signature. Pigment Cell Res. 2006;19(4):290-302.

6. Tsoi J, Robert L, Paraiso K, Galvan C, Sheu KM, Lay J, et al. Multi-stage Differentiation Defines Melanoma Subtypes with Differential Vulnerability to Drug-Induced Iron-Dependent Oxidative Stress. Cancer Cell. 2018;33(5):890-904 e5.

7. Andrews MC, Oba J, Wu CJ, Zhu H, Karpinets T, Creasy CA, et al. Multi-modal molecular programs regulate melanoma cell state. Nat Commun. 2022;13(1):4000.

8. Wouters J, Kalender-Atak Z, Minnoye L, Spanier KI, De Waegeneer M, Bravo Gonzalez-Blas C, et al. Robust gene expression programs underlie recurrent cell states and phenotype switching in melanoma. Nat Cell Biol. 2020;22(8):986-98.

9. Verfaillie A, Imrichova H, Atak ZK, Dewaele M, Rambow F, Hulselmans G, et al. Decoding the regulatory landscape of melanoma reveals TEADS as regulators of the invasive cell state. Nat Commun. 2015;6:6683.

10. Rambow F, Rogiers A, Marin-Bejar O, Aibar S, Femel J, Dewaele M, et al. Toward Minimal Residual Disease-Directed Therapy in Melanoma. Cell. 2018;174(4):843-55 e19.

11. Bedia C, Casas J, Garcia V, Levade T, Fabrias G. Synthesis of a novel ceramide analogue and its use in a high-throughput fluorogenic assay for ceramidases. Chembiochem. 2007;8(6):642-8.
